# Evaluation of the Effects of *Mitragyna speciosa* Alkaloid Extract on Cytochrome P450 Enzymes Using a High Throughput Assay

**DOI:** 10.3390/molecules16097344

**Published:** 2011-08-29

**Authors:** Wai Mun Kong, Zamri Chik, Murali Ramachandra, Umarani Subramaniam, Raja Elina Raja Aziddin, Zahurin Mohamed

**Affiliations:** 1 Department of Pharmacology, Faculty of Medicine, University of Malaya, 50603 Kuala Lumpur, Malaysia; E-Mails: zamrichik@um.edu.my (Z.C.); zahurin@um.edu.my (Z.M.); 2 Aurigene Discovery Technologies (M) Sdn. Bhd, Malaysia, 57000 Bukit Jalil, Kuala Lumpur, Malaysia; E-Mail: umarani_s@aurigene.com; 3 Aurigene Discovery Technologies Limited, Bangalore 560100, Karnataka, India; E-Mail: murali_r@aurigene.com; 4 Department of Pathology, Hospital Kuala Lumpur, 50586 Kuala Lumpur, Malaysia; E-Mail: rajaelina@yahoo.com

**Keywords:** herb-drug interactions, cytochrome P450 (CYP), *Mitragyna speciosa*, *in vitro*, alkaloids

## Abstract

The extract from *Mitragyna speciosa* has been widely used as an opium substitute, mainly due to its morphine-like pharmacological effects. This study investigated the effects of *M. speciosa* alkaloid extract (MSE) on human recombinant cytochrome P450 (CYP) enzyme activities using a modified Crespi method. As compared with the liquid chromatography-mass spectrometry method, this method has shown to be a fast and cost-effective way to perform CYP inhibition studies. The results indicated that MSE has the most potent inhibitory effect on CYP3A4 and CYP2D6, with apparent half-maximal inhibitory concentration (IC_50_) values of 0.78 µg/mL and 0.636 µg/mL, respectively. In addition, moderate inhibition was observed for CYP1A2, with an IC_50_ of 39 µg/mL, and weak inhibition was detected for CYP2C19. The IC_50_ of CYP2C19 could not be determined, however, because inhibition was <50%. Competitive inhibition was found for the MSE-treated CYP2D6 inhibition assay, whereas non-competitive inhibition was shown in inhibition assays using CYP3A4, CYP1A2 and CYP2C19. Quinidine (CYP2D6), ketoconazole (CYP3A4), tranylcypromine (CYP2C19) and furafylline (CYP1A2) were used as positive controls throughout the experiments. This study shows that MSE may contribute to an herb-drug interaction if administered concomitantly with drugs that are substrates for CYP3A4, CYP2D6 and CYP1A2.

## 1. Introduction

*Mitragyna speciosa* Korth is a tropical herb plant belonging to the family Rubiaeceae found mainly in Southeast Asian countries such as Thailand and Malaysia. It is known as “Biak-biak” or “Ketum” in Malaysia, and as “Kratom” in Thailand [1]. Thai and Malaysian natives traditionally consume the leaves by chewing, smoking or drinking them as tea, mainly for the stimulant and euphoric effect [2]. In human and animal studies, the leaves have been reported to possess morphine-like properties, including antitussive, anaesthetic, antinociceptive, analgesic and stimulative effects [3]. Since the nineteenth century, Kratom has been widely used as an opium substitute during opium withdrawal, as well as for pain relief. However, addiction and signs of opioid abstinence syndrome, such as irritability, yawning, rhinorrhoea, myalgias, diarrhoea, tremor, nausea, nystagmus and arthralgia, have also been reported [2], thereby limiting its use. 

Although the pharmacological effects of Kratom in humans and animals have been well established, the doses required to produce stimulation, analgesia and toxicity in humans still remain poorly defined. Abuse of the plant by drug addicts, however, has caused major concerns in Malaysia and Thailand, and consequently, the Kratom plant has been listed as a controlled item in Malaysia, Thailand and Australia. In other parts of the world, Kratom is currently not strictly regulated, and access to Kratom through the Internet has led to significant drug abuse problem among the Western population seeking self-treatment of opioid withdrawal and chronic pain [2,4]. 

Cytochrome P450 (CYP) is the major family of enzymes involved in metabolism of drugs, toxicants and endogenous compounds. Metabolism is generally regarded as a protective mechanism *via* the production of inert metabolites, but it can also cause toxicity by activation of pro-drugs to active metabolites. Members of the CYP subfamilies exhibit relatively strict specificities in the metabolism of xenobiotics. The CYP1, CYP2 and CYP3 subfamilies are responsible for the metabolism of >90% of commercially available drugs [5]. Preliminary understanding of the metabolism of a new chemical entity and its affinity to certain metabolizing enzymes is helpful during drug development to avoid undesirable drug-drug interactions that may lead to changes in the rate of drug metabolism and potentially contribute to drug toxicity [6]. For example, ketoconazole and quinidine are well-known CYP3A4 inhibitors that can induce life-threatening heart rhythm disorders when co-administered with other substrates of CYP3A4, such as erythromycin [7,8]. In 2006, the U.S. Food and Drug Administration drafted an industry guideline on drug interaction studies to reflect the importance of studying drug metabolism when assessing the safety and effectiveness of new drugs [9].

Many methods are available to measure enzyme activity in order to avoid pharmacokinetic drug-drug interaction due to CYP enzyme inhibition. These methods can be categorized according to the experimental system and screening strategies [10]. During the drug development process, the fluorescence measurement technique has the advantage of being less time-consuming and more cost-effective. For this method, the pro-fluorescent substrate is broken down to a fluorescent product by CYP enzymes and detected directly using a fluorescence plate reader. With the development of sensitive liquid chromatography-tandem mass spectrometry (LC-MS/MS) instruments, the analysis of metabolite generation from unlabelled drug substrates has become more efficient and highly specific. However, compared with the results of the fluorescence method, the productivity of drug-drug interaction assessment with LC-MS/MS is relatively low. Moreover, the requisite LC-MS/MS instrumentation is costly. 

The objective of this study was to evaluate the effect of *M. speciosa* alkaloid extract (MSE) on CYP enzymes by using novel high throughput *in vitro* fluorescent P450 assays. MSE was tested for its effect on CYP3A4, CYP2D6, CYP1A2 and CYP2C19 to determine the potential risk for causing interactions with other therapeutic products. 

## 2. Results and Discussion

### 2.1. Determination of Optimal Incubation Time

The calibration curves were prepared at different incubation times. Optimal incubation times were determined when good linearity was obtained in which the r^2^ value approached 1.0. The optimal incubation time for CYP3A4, CYP2D6 and CYP2C19 was 30 min, whereas the optimal incubation time for CYP1A2 was 20 min. For all CYP inhibition assays, the formation of fluorescent metabolites was proportional to the substrate concentration and incubation time. To determine the K_m_ and V_max_ values, we chose the optimal incubation time obtained from the previous experiment.

### 2.2. Determination of K_m_ and V_max_ Values

The assay was designed according to the Crespi method in that K_m_ and V_max_ values were determined using 10 substrate concentrations generated by 3× dilutions (1–300 µM). At a substrate concentration above 300 µM, a precipitation was observed that affected the fluorescence measurement. The K_m_ values for CYP3A4, CYP2D6, CYP1A2 and CYP2C19 were 48.94, 1.016, 23.69 and 4.627 µM, respectively. The K_m_ values estimated in this study were similar to those reported by Crespi *et al.* [11]. The V_max_ values for CYP3A4, CYP2D6, CYP1A2 and CYP2C19 were found to be 2,343, 47.18, 20,307 and 2,087 RFU product/min/pmol P450, respectively. The velocity of the reaction showed a hyperbolic pattern and approached the maximum velocity with increasing substrate concentration. The K_m_ values of the standard inhibitor are summarized in Table 1. The substrate concentration used for the subsequent assay was close to the apparent K_m_ values so that competitive inhibition could be detected with comparable efficiency.

### 2.3. Determination of IC_50_ for Standard Inhibitors 

The IC_50_ values for each of the P450 enzymes were determined by using a single concentration of enzyme and substrate that had been optimized previously. The standard inhibitor IC_50_ values were then compared with those in the literature and with the database of Aurigene Discovery Technologies (Table 1). IC_50_ value determinations were performed with quinidine (CYP2D6), ketoconazole (CYP3A4), tranylcypromine (CYP2C19) and furafylline (CYP1A2) as positive controls. Quinidine, ketoconazole, tranylcypromine and furafylline are both specific and potent inhibitors of specific liver CYP enzymes [9,11,12]. The IC_50_ values for the standard positive inhibitors in this study were very close to the reference values, indicating that the condition of the *in vitro* system was suitable for subsequent pharmacokinetic studies [13].

### 2.4. Determination of IC_50_ for MSE

**Figure 1:**
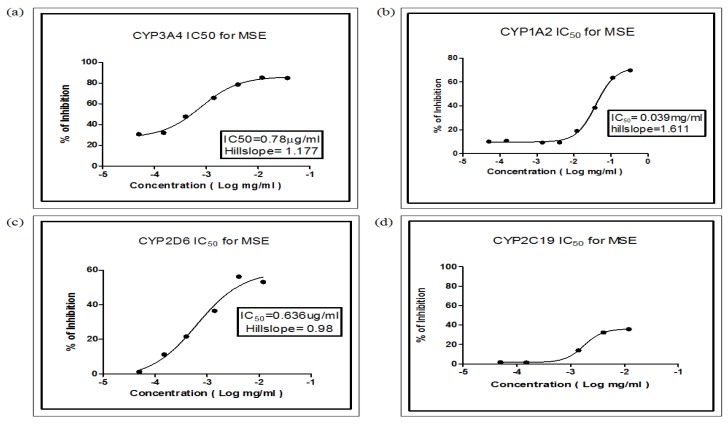
Percentage of inhibition of MSE on (**a**) CYP3A4; (**b**) CYP1A2; (**c**) CYP2D6; and (**d**) CYP2C19 activities after incubating at respective optimal incubation times. Two different concentrations (2 µM and 0.2 µM) of positive control (quinidine [CYP2D6], ketoconazole [CYP3A4], tranylcypromine [CYP2C19] and furafylline [CYP1A2]) were used throughout the assay.

Standard positive inhibitors and MSE were added separately to the *in vitro* drug-metabolizing systems at varying concentrations (2–10 µM for standard positive inhibitors and 0.05–100 µg/mL for MSE). The CYP-dependent inhibition was observed only at doses lower than 250 µg/mL MSE. High concentrations of MSE were found to interfere with the fluorescence measurement [1]. We found that MSE was a strong inhibitor of CYP3A4 and CYP2D6 with an IC_50_ of 0.78 µg/mL and 0.636 µg/mL, respectively. MSE appears to inhibit CYP1A2 moderately with an IC_50_ of 39 µg/mL. The IC_50_ of CYP2C19 was not determined, however, because inhibition was <50%. The results are summarized in Table 2 and Figure 1.

### 2.5. Determination of K_i_ Values and Modes of Inhibition for MSE

**Figure 2:**
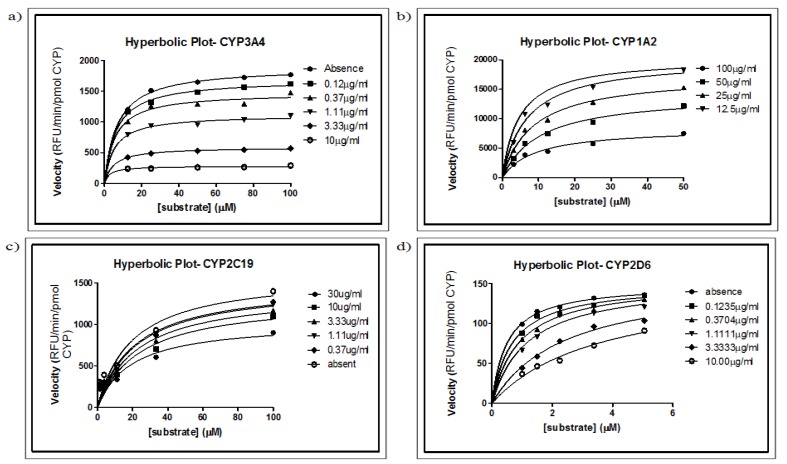
K_i_ determination using the non-linear regression method. Direct plot reaction velocity against different concentrations of substrate in the absence or presence of different concentrations of *Mitragyna speciosa* extract. Increasing the *M. speciosa* extract concentration resulted in reduced apparent V_max_ and increased apparent K_m_ for CYP3A4, CYP1A2 and CYP2C19. For CYP2D6, V_max_ remained constant but the K_m_ value was increased (not shown).

**Figure 3:**
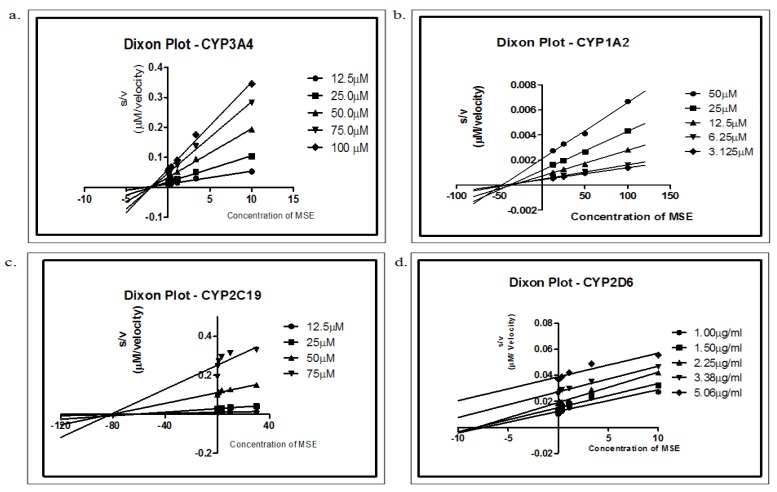
Dixon plot of substrate concentration/velocity (*s/v*) against *i* (*M. speciosa* extract) concentration for each of the substrate concentrations tested. The resulting lines intercept at a point corresponding to K_i_. Each point was the mean of triplicate determinations.

The most important measurement for a compound as an indication of its inhibitory potency is K_i_, the inhibition constant. It is an indication of the affinity of a compound for an enzyme. Various concentrations of substrates were incubated with the respective CYP isoforms in the absence and presence of different concentrations of MSE. The K_i_ and modes of inhibition for MSE in different CYP activities are summarized in Table 2. MSE showed non-competitive inhibition for CYP3A4, CYP1A2 and CYP2C19, in which the apparent K_m_ was increased, together with a decrease in V_max_. The reduction of the “active” enzyme results in a decrease of V_max_. However, MSE was a competitive inhibitor for CYP2D6, as shown by the increasing values of K_m_, with V_max_ remaining unchanged (Figure 2). The Dixon plot *s/v* versus concentration of MSE was plotted to differentiate the mode of inhibition (Figure 3).

Since the 1980s, studies on specific forms of CYP using *in vivo* and *in vitro* systems have attracted the attention of researchers because of a most interesting characteristic of CYP: substrate specificity and inhibitor selectivity. CYP2D6 is relatively specific for metabolizing positively charged molecules with a basic nitrogen atom. CYP1A2, on the other hand, is involved in the metabolism of polyaromatic hydrocarbons, and CYP2C9 metabolizes weakly anionic molecules [15]. Most organic compounds can therefore be metabolized by CYP enzymes. It should be kept in mind that the kinetic characteristics of enzymes are important for the metabolism and clearance of drugs [12]. In this study, CYP1A2 showed a relatively high reaction rate compared with other CYPs, whereas CYP2D6 showed the lowest reaction rate; CYP3A4 and CYP2C19 had a moderate rate of reaction. There was a relatively large difference between the V_max_ values found in this study from those reported in the literature [11,12] This finding may be due to different sources of human liver microsomes used such as P450 expression cell lines and baculovirus infected insect cells (BTI-TN-5B1-4) [10,11]. Differences in enzyme expression systems, including proteins, lipids, co-enzyme concentrations, ratios of reductase and cytochrome b5, phospholipid composition of microsomes and incubation conditions adopted in different laboratories, may also bring about significant changes in the V_max_ and K_m_ values [10]. 

**Figure 4:**
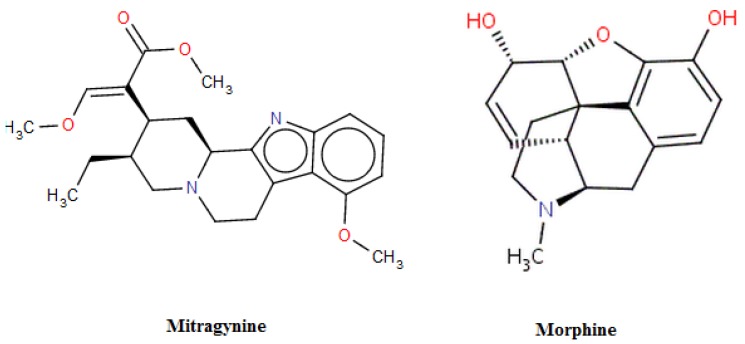
Chemical structure of mitragynine and morphine.

Mitragynine, reported to be the most abundant alkaloid in *M. speciosa* extracts [16], has some morphine-like properties such as antinociceptive, antitussive, antimalarial and antidiarrheal effects [17]. Interestingly, mitragynine is structurally different from morphine (Figure 4) yet the two produce similar pharmacological effects. The chemical structure of mitragynine contains an indoloquinolizidine moiety with a methoxy group at the C-9 position. As reported, the antinociceptive effect of the leaves was mediated by µ- and δ-opiate receptors which have a similar mechanism of action as morphine. However, the finding of Matsumoto *et al*. showed that 7-hydroxymitragynine has stronger antinociceptive effects as compared to morphine and mitragynine. The introduction of hydroxy group at the C-7 position has been proposed to have stronger affinity to µ- and k-opiate receptor [18]. Seven metabolites had been identified after the Phase I metabolism hydrolysis of the methyl-ester group in position 16, 9 and 17; while four metabolites were found in the Phase II metabolism of mitragynine in human and animal [19]. Anyhow, the effects of those metabolites still remains unknown. Due to the unique hydrophobic properties of plant alkaloids, they easily pass through the blood brain barrier and affect the opiate, noradrenaline, dopamine and serotonin receptors. Other active constituents found in the extract, such as speciofoline, rhychophylline and stipulatine, are suspected to contribute to some extent to the herb-drug interaction [20]. However, the active substances of *M. speciosa* responsible for substrate metabolism in this study remain unknown, and thus underestimation of the total inhibitory potential of the compound may have occurred. Nonetheless, the alkaloid extract in our study may provide fundamental and representative data on the isoforms examined because people usually consume the herb as a whole leaf instead of in the form of a particular active ingredient. 

A comparison of our results for the IC_50_ of MSE with those of Hanapi *et al.* suggests that our study had a similar pattern of inhibition but with a stronger inhibitory effect [6]. Our results appear to show a 100-fold stronger inhibition for CYP3A4 and a 6-fold stronger inhibition for CYP2D6 compared with the results from the Hanapi *et al*. study [6]. On the other hand, the reported MSE-induced CYP2C19 inhibition effect in both studies was considered low. However, the IC_50_ value of CYP2C19 in Hanapi *et al*. was relatively high compared with our results [6]. These findings may be due to the variability of working with natural products. Unlike conventional single active compounds, natural products are complex mixtures containing various chemical substrates that can vary as a result of environmental factors such as climate, growth conditions, harvest and storage conditions [6,15]. In addition, variations in the manner of preparation and the extraction methods can also contribute to data variability. 

In this study, MSE was found to be a non-competitive inhibitor for CYP3A4, CYP1A2 and CYP2C19, with K_i_ values of 1.526, 18.57 and 84.88 µg/mL, respectively. Non-competitive inhibition is characterized by binding of the inhibitor to the side other than the active site (allosteric site) of the free enzyme or of the enzyme substrate complex. The presence of the inhibitor causes a change in the structure of the enzyme so that it no longer binds to the substrate or catalyzes product formation. An increased K_m_ value and decreased maximum enzyme reaction rate (V_max_) was observed, as shown in Figure 2. Non-competitive inhibition can be easily distinguished from other types of inhibition by identifying similar K_i_ values in both the 1*/v* against *i* and *s/v* against *i* graphs. In addition, another characteristic of non-competitive inhibition is that the intersections are found on the *i* axis in both graphs [21].

In competitive inhibition, the substrate competes with the inhibitor for binding to the active site of the enzyme. Therefore, inhibition can be overcome at sufficiently high substrate concentrations, with the V_max_ remaining unaffected. Moreover, the condition K_i_ = ∞ (the lines are parallel) was found in the Dixon plot (*s/v* against *i*), whereas an intersection was found in the conventional Dixon plot *1/v* against *i* [21]. This finding indicated that MSE was a competitive inhibitor for CYP2D6. No report presently exists regarding the effect of MSE on the mode of inhibition of the CYP enzyme; hence, no comparison could be made with the present data.

Since MSE is a potent CYP3A4 and CYP2D6 inhibitor, co-administration of drugs and herbs that are metabolized by the CYP enzymes CYP3A4 and CYP2D6 will have the potential to abolish metabolic clearance *in vivo*, resulting in unwanted toxic effects. Hence, the study of herbs as potential drug inhibitors is important for minimizing the unwanted consequences of herb-drug interactions. The potential of the extract of *M. speciosa* to affect drug clearance and deposition may increase if used in combination with one or more drugs that are substrates for CYP3A4, CYP2D6 and CYP1A2; with other herbs that are known substrates of CYP3A4; and with other isoforms of these CYP enzymes, such as St. John’s wort, garlic, gingko, ginseng and grapefruit, which have been reported to have CYP inhibition properties [16,22]. Hence these substrates can potentially enhance or inhibit the effects of *M. speciosa* [15]. High doses of *M. speciosa* extract might be taken by chronic users because of tolerance effects. Since MSE showed a potent inhibitory effect on CYP3A4 and CYP2D6, the two enzymes involved in the metabolism of most drugs, with IC_50_ and K_i_ values lower than 20 µg/mL [14], it is necessary for patients and doctors to be aware of potential drug interactions when MSE is co-administered with other medications.

## 3. Experimental

### 3.1. Chemicals

Recombinant human CYP3A4, CYP2D6, CYP1A2 and CYP2C19 enzymes; marker substrates 3-[2-(*N,N*-diethyl-*N*-methylammonium)ethyl]-7-methoxy-4-methylcoumarin (AMMC), 7-benzyloxy-4-(trifluoromethyl)-coumarin (BFC) and 7-hydroxy-4-(trifluoromethyl)-coumarin (HFC); and reduced nicotinamide adenine dinucleotide phosphate (NADPH) regeneration system (NRS) were purchased from Gentest Corporation (Woburn, MA, USA), while all the other chemicals and standard references, including 3-cyano-7-ethoxycoumarin (CEC), quinidine, ketoconazole, tranyl-cypromine and furafylline, were purchased from Sigma Aldrich (St. Louis, MO, USA).

### 3.2. Plant Extraction

Fresh leaves of *M. speciosa* Korth were collected from the Kangar forest (Perlis State, Malaysia), which is close to the national border between Malaysia and Thailand. A methanol-chloroform extraction method was used to extract the alkaloids. The leaves (5 kg) were dried and soaked in 4 L methanol for three days. The methanol was filtered and the filtrate was evaporated using a rotary evaporator. This extraction and evaporation procedure was repeated three times. After that, 1 part of crude methanol extract was redissolved in around 35 parts of 1:9 acetic acid-distilled water and then washed with an adequate amount of hexane. The acidic layer was made alkaline to pH 9 using ammonia hydroxide and extracted with chloroform. The collected organic layer was filtered through anhydrous sodium sulphate, and the filtrate was concentrated using a rotary evaporator to obtain 5 g of crude alkaloid extract, which was then dissolved in dimethyl sulfoxide (DMSO) and the presence of alkaloid was confirmed using the Dragendorff test [23].

### 3.3. Fluorometric Enzyme Inhibition Assays

#### 3.3.1. Time and Concentration Linearity

The fluorescence readings of the corresponding metabolites were measured at 10, 20, 30, 45, 60 and 90 min after adding the NRS solution. K_m_ values that had been established by Crespi *et al*. were used as the substrate concentrations in this assay [11]. Optimal incubation times were calculated from the linearity of the graph. 

#### 3.3.2. Determination of Km and Vmax Values

Reactions were carried out with 10 different substrate concentrations: 1–200 µM BFC for CYP3A4, 0.04–100 µM AMMC for CYP2D6, 0.13–100 µM CEC for CYP1A2 and 0.23–300 µM CEC for CYP2C19, in three-fold dilutions with respective buffer. The stock concentration was 20 mM for all the substrates. The previously determined optimal incubation times were used throughout the experiment. K_m_ and V_max_ values for each of the CYP enzymes were determined by using Michealis-Menten plots. The concentration of DMSO did not exceed 0.2% and that of ACN did not exceed 2%. DMSO and acetonitrile (ACN) have been known to be potent inhibitors of CYP enzymes. 

#### 3.3.3. Half-Maximal Inhibitory Concentration (IC_50_) Determination of Inhibitors and Plant Extracts 

The assays were performed by using the modified Crespi method [11]. Incubations were conducted in a total reaction volume of 150 µL in 96-well Fluorolux^TM^ HB black, flat-bottom microplates from Dynex (Chantilly, VA, USA). The final concentrations of the plant extracts (0.05–1000 µg/mL) and positive controls (0.005–100 µM) were prepared by using a 3× dilution method. NRS was prepared by using 3.3 mM of glucose-6-phosphate (G6P), 0.06 U of glucose-6-phosphate dehydrogenase (G6PDH), 3.3 mM of MgCl_2_ and 1.3 mM of nicotinamide adenine dinucleotide phosphate (NADP^+^). The reaction was initiated by addition of NRS (20 µL) into a mixture of pre-warmed enzyme/substrate mix (30 µL), test compound (20 µL) and buffer (80 µL), followed by 30 min of incubation at 37 °C. Following that, the reactions were terminated by addition of stop solution (75 µL, 4:1 ACN: 0.5 M Tris base). The released fluorescence was scanned using a fluorescence plate scanner, VICTOR^TM^ X5, Perkin Elmer (Waltham, MA, USA), at the optimal wavelength of the metabolite. Positive and negative controls were run with every assay. Data were exported and analyzed with PRISM software (version 5.04). IC_50_ values were calculated using the relative IC_50_ determination. The results are presented as the mean of three replicates for at least two independent experiments and the protocol is summarized in Table 3.

#### 3.3.4. Determination of Ki Values and Modes Of Inhibition

The apparent inhibition constant (K_i_) values were further determined for concentrations of substrates ranging from 3.125 to 100 µM at different concentrations of MSE (0.125–300 µg/mL). Lineweaver-Burk plots, Dixon plots and secondary reciprocal plots were plotted to determine the K_i_ values and mode of inhibition.

## 4. Conclusions

The modified Crespi CYP inhibition method used in this study is a relatively fast and cost-effective way to perform a CYP inhibition assay. In this study, *M. speciosa* alkaloid extract inhibited CYP enzymes with varying degrees of potency. From the K_i_ and IC_50_ values, the results showed that MSE is a potent inhibitor of CYP3A4 and CYP2D6 (K_i_ and IC50 values ≤ 20 µg/mL), implying a high potential risk of herb-drug interactions, especially when patients consume large amounts of extract. The extract was found to moderately inhibit CYP1A2 (IC_50_ values 20–100 µg/mL) but there was no significant inhibition of CYP2C19 (<50% inhibition at the highest concentration of MSE). Competitive inhibition was observed for CYP2D6 and non-competitive inhibition was found for the other CYP isoforms. At present, the active constituent responsible for inhibition of the CYPs remains unknown. Further work is required to identify the inhibition properties of each of the active constituents of *M. speciosa* extracts.

## Figures and Tables

**Table 1:** K_m_ and IC_50_ values of the standard inhibitors reported in the literature.

Cytochrome/Substrate	This study			Reference	
	K_m_ (µM)	IC_50_ (µM)		K_m_ (µM) ^a^	IC_50_ (µM) ^a,b^
3A4/BFC	48.94	0.045		50	0.06 ± 0.016 ^b^ 0.09 ± 0.03 ^a^	
2D6/AMMC	1.016	0.005		1.5	0.0069 ± 0.0006 ^b^ 0.5 ± 0.2 ^a^	
1A2/CEC	23.69	0.755		25	0.58 ± 0.05 ^b^ 1.4 ± 0.3 ^a^	
2C19/CEC	4.627	2.511		5	2 ± 0.5 ^b^ 3.2 ± 1.2 ^a^	

^a^ Reference from database of Aurigene Discovery Technologies, Ltd.; ^b^ Donato *et al*. [13]. Data were expressed in standard error of the mean (S.E.M).

**Table 2:** Summary of IC_50_, K_i_ values and inhibition mode of MSE on CYP activities. ^a^ Ki values are derived from secondary plots of slopes taken from double reciprocal plots versus MSE concentrations. The compounds were classified as potent (IC50 ≤ 20 µg/mL or ≤10 µM); moderate (IC_50_ from 20 to 100 µg/mL or 10 to 50 µM) or weak (IC_50_ from ≥100 µg/mL or ≥50 µM) [14]. Data are the average values of duplicate determinations; ^b^ Nd, not determined.

Cytochrome P450	IC_50_ (µg/mL)	K_i_ (µg/mL)	Mode of inhibition
3A4/BFC	0.78	1.526 ^a^	Non competitive
2D6/AMMC	0.636	2.6 ^a^	Competitive
1A2/CEC	39	18.57 ^a^	Non competitive
2C19/CEC	Nd ^b^	84.88 ^a^	Non competitive

**Table 3:** Summary of the components of the fluorometric enzyme inhibition assays.

	CYP3A4	CYP1A2	CYP2C19	CYP2D6
**Substrate (final)**	BFC: 50 µM	CEC: 5 µM	CEC: 25 µM	AMMC: 1.5 µM
**Enzyme**	1.0 pmol/well	0.5 pmol/well	0.5 pmol/well	1.5 pmol/well
**Standard inhibitor**	Ketoconazole	Furafylline	Tranylcypromine	Quinidine
**Phosphate buffer**	200 mM	100 mM	50 mM	100 mM
**Fluorescence Filter**	Ex: 409 nm	Ex: 409 nm	Ex: 409 nm	Ex: 390 nm
	Em: 530 nm	Em: 460 nm	Em: 460 nm	Em: 460 nm
**Temperature**	30 °C	25 °C	30 °C	30 °C
**Incubation time**	30 min	20 min	30 min	30 min

## Abbreviations

MSE, *Mitragyna speciosa* alkaloid extract; CYP, cytochrome P450; AMMC, 3-[2-(*N,N*-diethyl-*N*-methylammonium)ethyl]-7-methoxy-4-methylcoumarin; BFC, 7-benzyloxy-4-(trifluoromethyl)-coumarin; HFC, 7-hydroxy-4-(trifluromethyl)-coumarin; NRS, NADPH regeneration system; CEC, 3-cyano-7-ethoxycoumarin; DMEM, Dulbecco’s modified Eagle medium; DMSO, dimethyl sulfoxide; G6P, glucose-6-phosphate; G6PDH, glucose-6-phosphate dehydrogenase; NADP+ nicotinamide adenine dinucleotide phosphate; RFU, relative fluorescence units.
